# Automated diagnosis of plus form and early stages of ROP using deep learning models

**DOI:** 10.1038/s41598-026-37064-2

**Published:** 2026-02-04

**Authors:** Mahdi Vahidmoghadam, Parisa Ghorbani, Mohammad Javad Ahmadi, Esmaeil Asadi Khameneh, Babak Tavassoli, Hamid D. Taghirad

**Affiliations:** 1 Applied Robotics and AI Solutions (ARAS), Faculties of Electrical and Computer Engineering, K.N. Toosi University of Technology, Tehran, Iran; 2 Farabi Eye Hospital, Tehran University of Medical Sciences, Tehran, Iran; 3 Faculty of Electrical Engineering, K. N. Toosi University of Technology, Tehran, Iran

**Keywords:** ROP, Plus disease, Stage of ROP, Artificial intelligence, Transfer learning, Blood vessels segmentation, Biomarkers, Computational biology and bioinformatics, Diseases, Health care, Medical research

## Abstract

Retinopathy of Prematurity (ROP) represents a critical ophthalmological pathology affecting premature infants, with established associations to low birth weight (BW) and early gestational age (GA). Elevated risk of severe ROP, which can result in irreversible vision loss, is observed in infants exhibiting lower BW and GA. This research investigates the development of an automated diagnostic system designed to classify Plus disease, a marker of abnormal retinal vascularity, and ROP staging, a determinant of disease progression. Specifically, the model facilitates binary classification of Plus disease (Plus/Normal) and multi-class classification of ROP stage (Stage 0, 1, 2, 3) using a meticulously curated dataset of retinal fundus images. The proposed model demonstrates high diagnostic accuracy, achieving 0.996 for Plus disease detection and 0.98 for ROP stage classification. These results suggest potential clinical utility for automated ROP screening methodologies in supporting timely diagnosis and intervention in similar settings, pending multi-center validation, which could help reduce the incidence of vision impairment in preterm populations.

## Introduction

Retinopathy of Prematurity (ROP) is an eye disease involving abnormal blood vessel growth in the developing retina of preterm infants. Established risk factors for development of ROP include reduced gestational age (GA) and low birth weight (BW)^1^. The development of ROP occurs in two distinct phases. Initially, the transition from the intrauterine to extrauterine environment disrupts normal retinal vascular development, resulting in retinal hypoxia. Subsequently, this hypoxic state triggers a proliferative phase marked by abnormal vascularization and glial cell proliferation, ultimately contributing to disease progression and the potential for significant visual impairment^2^.

Epidemiological studies demonstrate a significant correlation between gestational age and incidence and severity of ROP. Infants born before 32 weeks of gestation exhibit an ROP development rate of approximately 36.5%, with 10.15% progressing to severe ROP. In contrast, infants born after 32 weeks of gestation have a ROP development rate of 27.4%, with a lower progression rate to severe ROP, specifically 3.6%^3^. The significant prevalence of ROP, particularly among preterm infants, underscores the critical importance of early identification and timely clinical intervention. ROP remains a leading cause of childhood blindness, resulting in an estimated 50,000 cases of permanent visual impairment worldwide each year. The burden of this condition is predominantly concentrated in Latin America, Southeast Asia, and Eastern Europe^4^. In these regions, disparities in healthcare access, limited implementation of standardized screening programs, and a lack of trained ophthalmologists contribute to the exacerbation of ROP. Consequently, delayed diagnosis and treatment can cause severe ocular complications, including macular folds, retinal detachment, and irreversible blindness.

Global screening protocols for preterm infants represent a substantial economic burden on healthcare systems. For example, in the United Kingdom’s National Health Service, approximately 55 at-risk infants are screened to identify each infant that requires therapeutic intervention for ROP^5^. The substantial financial burden and interobserver variability associated with current screening practices underscore the imperative to develop cost-effective and standardized diagnostic methodologies for ROP. In addition, the global shortage of trained ophthalmologists, which is particularly acute in resource-constrained environments, impedes timely diagnosis and intervention. This, in turn, limits the efficacy of ROP screening programs in preventing visual impairment. In numerous regions, inadequate healthcare infrastructure, limited access to diagnostic imaging modalities, and a scarcity of qualified medical personnel pose significant barriers to effective ROP management^6^. Furthermore, access to surgical interventions for advanced ROP remains limited in numerous middle-income countries, particularly in Asia, where there is a critical shortage of pediatric retinal surgeons and anesthesiologists. Addressing these multifaceted challenges requires the development and implementation of scalable and efficient diagnostic methodologies, adaptable to diverse healthcare infrastructures.

Plus disease represents a critical prognostic indicator of the severity of ROP. This condition is characterized by increased dilation and tortuosity of the retinal blood vessels, exhibiting a strong association with severe ROP and an elevated risk of retinal detachment^7^. The timely identification of Plus disease is crucial to determine the necessity of therapeutic interventions, including laser photocoagulation or anti-VEGF injections, thus mitigating further retinal damage. The accurate staging of ROP is equally essential for assessing disease severity and guiding optimal management strategies. During the early stages of ROP (Stages 0–3), rigorous monitoring and clinical management can effectively prevent disease progression. However, progression to Stages 4 or 5 significantly increases the risk of irreversible blindness due to partial or complete retinal detachment^8^, and treatment options become substantially limited. The inherent complexity of ROP staging and diagnosis underscores the critical need for precise and standardized assessment methodologies.

Artificial intelligence (AI) is increasingly being applied across a broad spectrum of ophthalmic conditions, enhancing both the speed and accuracy of diagnosis. In diabetic retinopathy (DR), deep learning models have achieved diagnostic performance comparable to that of experienced ophthalmologists^9^. Similarly, in age-related macular degeneration (AMD), AI can now classify disease stages using fundus and OCT imaging^10^. Although glaucoma primarily involves the optic nerve, AI often utilizes retinal scans–particularly of the optic nerve head and RNFL–to identify early indicators^11^. AI has also shown promise in detecting genetic retinal disorders such as Stargardt’s disease and retinitis pigmentosa through analysis of diverse imaging datasets^12^. Hypertensive retinopathy is another area where AI demonstrates value by detecting subtle signs like arterial narrowing or microhemorrhages^13^. Interestingly, fundus images have even been used to predict cardiovascular risk, underscoring the retina’s broader diagnostic potential^14^. Beyond retinal diseases, AI contributes to other subspecialties as well. In cataract management, machine learning algorithms analyzing slit-lamp images can grade lens opacities and assist in surgical decision-making^15^. In corneal disorders like keratoconus, AI systems leveraging topography or tomography data are being developed to detect early changes and monitor progression^16^.

**Fig. 1:**
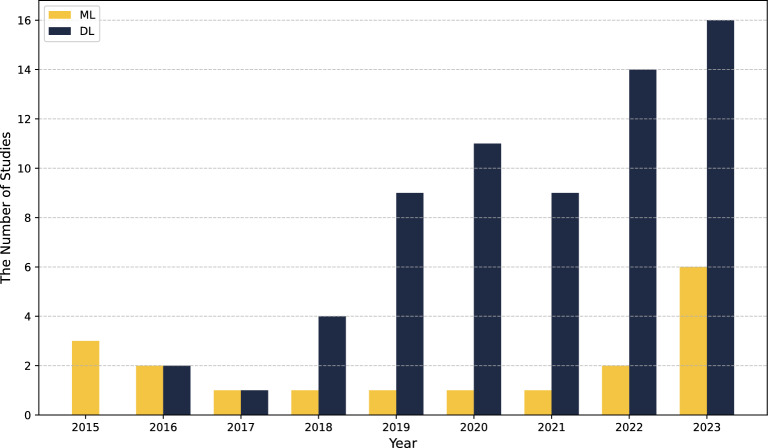
Bar chart showing the number of AI studies published in ROP detection^17^.

The integration of artificial intelligence (AI) into the diagnostic workflow for ROP has significantly advanced disease detection by improving accuracy, consistency, and screening efficiency. As depicted in Fig. 1, the application of various artificial intelligence methodologies in the diagnosis of ROP has also shown a notable increase. AI-based systems enable automated classification of Plus disease and ROP Stages, thereby reducing dependence on subjective clinical assessments and minimizing inter-observer variability. These AI-driven algorithms have the potential to improve access to expert-level diagnostic tools, especially in areas with a shortage of specialized ophthalmologists, and may ultimately help reduce vision loss in preterm infants, though multi-center validation is essential to confirm generalizability across diverse clinical environments.

Given the critical importance of an accurate and timely diagnosis of ROP, as discussed earlier, this study focuses on developing a highly accurate AI-based system to detect both the disease stage and the presence of Plus disease. As the structural patterns of retinal blood vessels play a crucial role in determining these conditions, the first step in our proposed pipeline involves segmentation of the retinal vasculature from fundus images. To achieve this, we train a specialized deep-learning model for vessel extraction. Additionally, to ensure robust performance on images with poor quality or noise, several preprocessing techniques, including denoising filters and contrast enhancement methods, are applied to improve the reliability of the segmentation process.

Following vessel segmentation, we train classification models to identify the different stages of ROP as well as the presence or absence of Plus disease. To determine the optimal input representation for these classification tasks, we evaluated multiple configurations: raw retinal images, segmented vessel maps, fused images combining fundus and vessel data, and a four-channel input format where the vessel map is integrated as an additional channel along with the standard RGB image. This multi-input strategy allows us to assess which representation yields the highest diagnostic accuracy. Furthermore, we experiment with various deep learning backbones to identify architectures that provide the best trade-off between performance and computational efficiency.

Finally, to gain deeper insight into the model’s decision-making process, we apply t-SNE to visualize high-dimensional feature embeddings from the final layers of the network, providing a qualitative assessment of class separability. In addition, we utilize Grad-CAM to highlight the most prominent regions in the input images that influence the model’s predictions for Stage and Plus classification. These visualization techniques not only support interpretability; but also help validate the clinical relevance of the focus areas of the model.

## Methods

### Dataset

The dataset used in this study for classification was collected at the University Hospital Ostrava and is publicly available. This dataset consists of retinal images obtained from 188 infants. Devices used for image collection include Clarity RetCam 3, Phoenix ICON, and Natus RetCam Envision.

A total of 6004 images were collected from these 188 infants, with different types of labels applied to them. The conditions examined in this study include the detection of plus disease and the classification of Stages of ROP. For Stage classification, a total of 880 images are used, which include 45 images from Stage 0, 252 images from Stage 1, 458 images from Stage 2, and 125 images from Stage 3. Also, for the detection of Plus disease, all 6004 images in the dataset are used, comprising 5,375 images labeled Normal and 629 images labeled Plus disease^18^. Ground-truth labels were sourced from validated ROP databases, where staging adhered strictly to ICROP guidelines, incorporating expert assessment of morphological features at the vascular-avascular junction, such as the demarcation line for stage 1 and ridge for stage 2 and extraretinal neovascular proliferation or flat neovascularization for stage 3. As shown in Table 1, the dataset was divided into three subsets: 80% of the images were allocated for training, 16% for validation, and 4% for testing. The training set was used for model training, the validation set for model validation and performance metric computation, and the test set for final model evaluation. The three subsets (training, validation, and test) were entirely independent, with no overlap in images, ensuring unbiased evaluation. Additionally, the test set samples used for Grad-CAM visualizations also contributed to the independent assessment of the model’s performance.

**Table 1:** Plus and Stage dataset distribution across training, validation, and test sets.

Disease	Total images	Train	Validation	Test
Plus	6004	4803	960	241
Stage	880	703	140	37

Furthermore, for the segmentation task and the extraction of blood vessels from retinal images, a total of three datasets were utilized: FIVES^19^ with 601 images, HRF^20^ with 30 images, and HVDROPDR^21^ with 50 images, resulting in a combined total of 681 retinal images.

### Deep learning algorithm

In this study, as previously mentioned, newborn retinal images are used for the diagnosis of various stages of ROP. Consequently, deep learning methodologies have been employed to facilitate an accurate diagnosis directly from these images. It should be noted that some prior research has explored the diagnosis of this condition using machine learning techniques, incorporating clinical information such as gestational age (GA) at birth and birth weight (BW) as key predictive features^22^.

The use of retinal images in conjunction with deep learning offers several key advantages over diagnostic approaches relying solely on clinical parameters and conventional machine learning. First, image-based analysis allows direct visualization and assessment of subtle pathological changes within the retina, which may not be readily apparent in clinical data alone. Deep learning models excel at automatically learning intricate features from these complex visual inputs, surpassing the need for manual feature engineering, often required in traditional machine learning. This capability enables the identification of nuanced patterns indicative of early ROP Stages or specific disease subtypes with potentially higher sensitivity and specificity.

Furthermore, while clinical data such as GA and BW provide valuable risk factors, they represent indirect indicators of disease development. Image analysis, on the other hand, offers a direct assessment of the target organ, potentially capturing the heterogeneity and dynamic progression of the ROP more effectively. Deep learning models trained on large datasets of retinal images can learn to recognize complex spatial and temporal patterns associated with disease progression, potentially leading to earlier and more accurate diagnoses compared to models relying solely on static clinical variables. This direct, data-driven approach has the potential to improve the timeliness of the intervention and ultimately lead to better visual outcomes for preterm infants at risk of ROP.

#### Segmentation and blood vessels extraction

As mentioned above, one of the key objectives of using artificial intelligence (AI) in the diagnosis of ROP is to achieve high accuracy in the classification of Plus disease and stages of the disease. This goal is contingent upon the extraction of relevant key features from retinal images. To this end, a segmentation model is used to extract retinal blood vessels. These extracted vessels are then used directly for the classification of Plus disease and, when combined with the original retinal images, used for Stage classification.

The rationale for the direct use of images of extracted blood vessels in the Plus disease classification lies in the strong dependence of this classification on retinal vascular characteristics, such as vessel tortuosity and dilation. Furthermore, the combination of extracted vessel images with the original retinal images improves the accuracy of ROP stage classification by providing a more comprehensive representation of the characteristics of the disease.

**Fig. 2:**
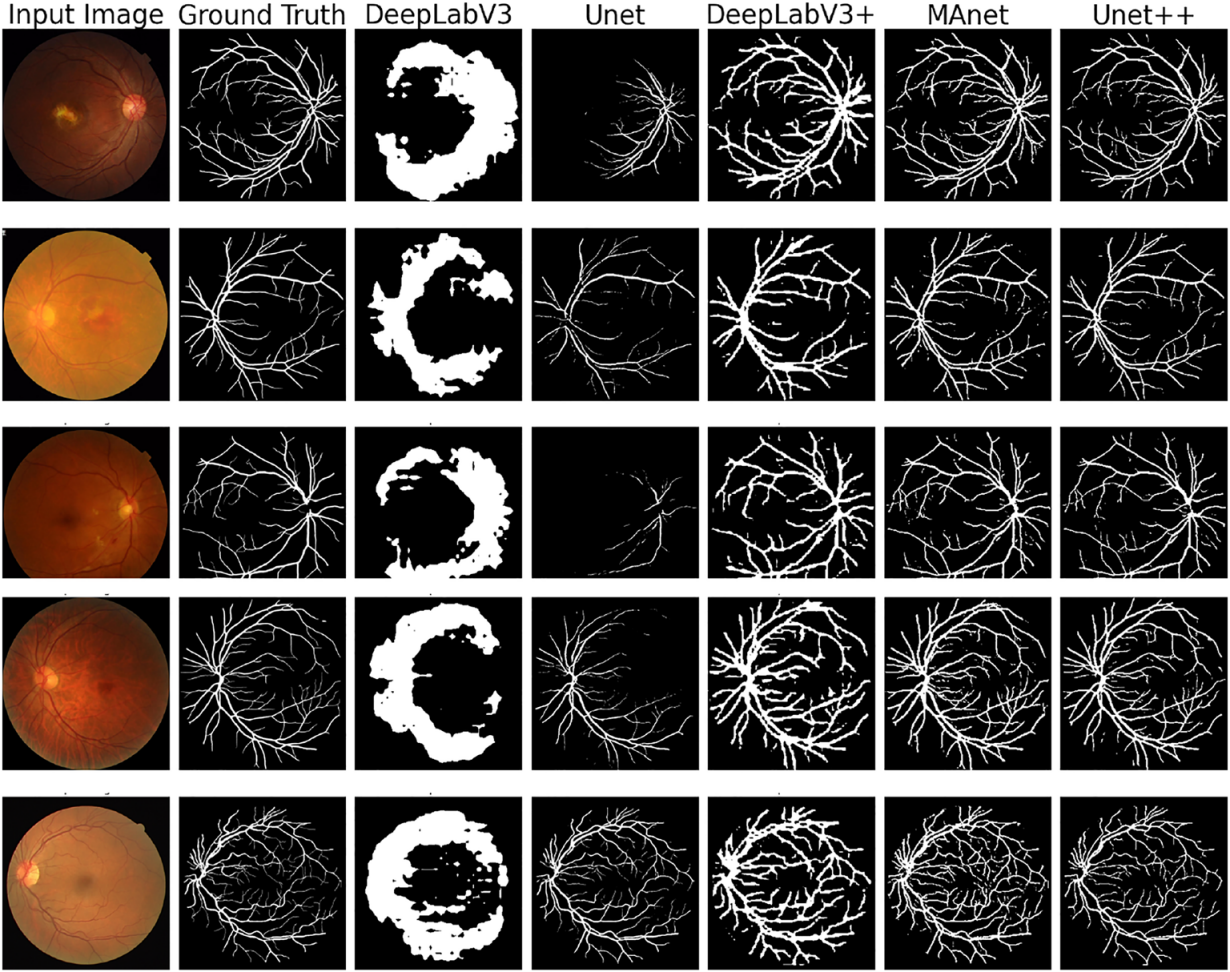
Visual comparison of retinal vessel segmentation results. From left to right: Input image, ground truth segmentation, and predictions from DeepLabV3, Unet, DeepLabV3+, MAnet, and Unet++ models.

To extract blood vessels from retinal images, we trained various models, including DeepLabV3^23^, DeepLabV3+^24^, U-Net^25^, U-Net++^26^, and MAnet^27^, to determine the most effective model for this task. The results obtained are presented in Table 2. As illustrated in Fig. 2, the U-Net++ model demonstrated the best performance in extracting retinal blood vessels. It should be noted that the MAnet model also showed relatively good performance; however, U-Net++ performed better, particularly in capturing fine details and terminal points of the vessels.

**Table 2:** Performance metrics evaluated from the segmentation models used for blood vessel segmentation on Validation set.

Model	Accuracy	Recall	IoU	Dice
DeepLabV3	0.9036	0.6228	0.4254	0.5967
DeepLabV3+	0.9451	0.7172	0.5315	0.6932
Unet	0.91	0.6621	0.4564	0.6381
MAnet	0.9749	0.8358	0.7453	0.8534
Unet++	**0.9766**	**0.8385**	**0.7562**	**0.8584**

Significant values are in [bold].

Furthermore, the performance of classification models is strongly dependent on the accurate extraction of all blood vessels present in the retinal images. Therefore, in this study, we have adopted an approach to ensure the proper extraction of vessels, particularly in cases where image quality is poor.

**Fig. 3:**
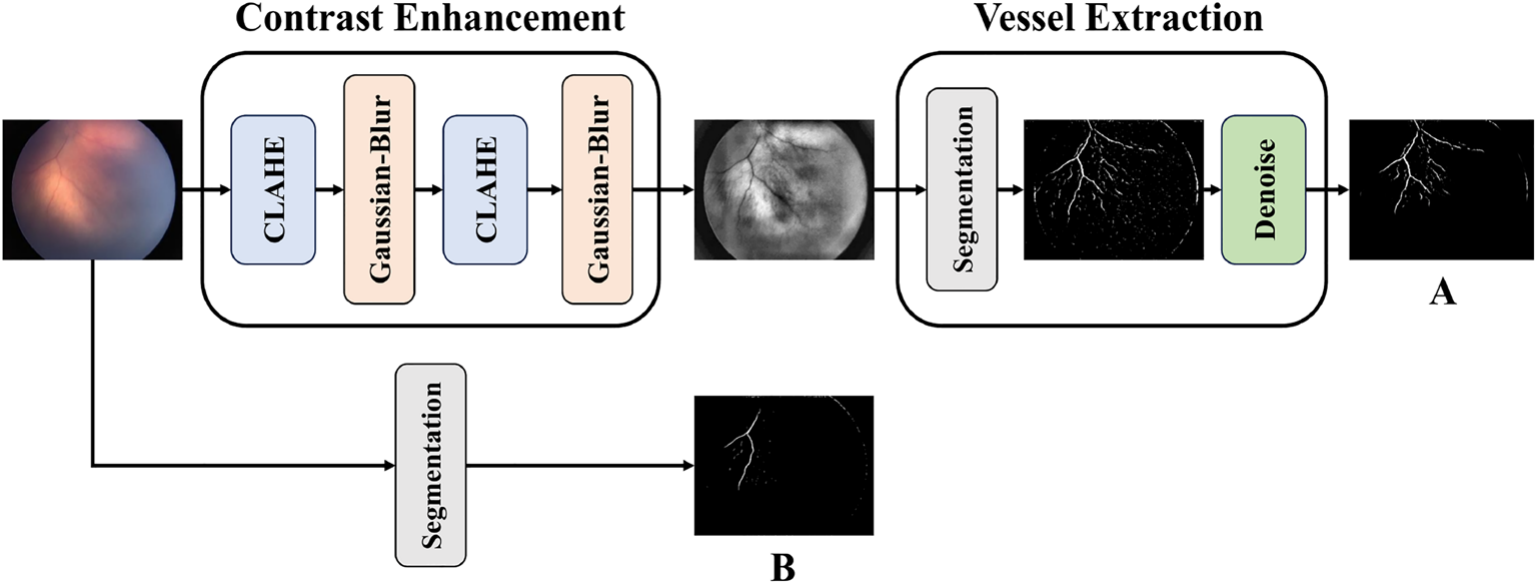
(**A**) In the proposed method for enhanced vessel extraction, a filter is applied twice to the image to improve detail extraction before being processed by the segmentation model. Due to noise in the model’s output, a noise reduction technique is then applied to refine the extracted vessels. (**B**) The retinal image is directly fed into the segmentation model, where vessel extraction is performed using the standard approach.

As illustrated in Fig. 3, this method first improves the contrast of the image using filters such as CLAHE^28^ (Contrast Limited Adaptive Histogram Equalization) and GaussianBlur, making blood vessels more distinguishable. The enhanced image is then reapplied to the original image to further improve vessel visibility. Next, this processed image is fed into the segmentation model, which extracts blood vessels from the retinal image. In the final step, noise reduction techniques are applied to the output of the segmentation model to eliminate unnecessary regions and refine the extracted vessels.

#### Stage and plus disease classification

This section will focus on the classification of the Plus disease and Stage conditions of ROP, which represent the primary objective of this study. To achieve the highest accuracy in the classification of these two critical states, we will investigate various input image modalities and different convolutional neural network (CNN) backbones for feature extraction from the retinal images. The aim is to identify the most effective combination for accurate and robust differentiation between Plus disease and the various Stages of ROP.

#### Class imbalance mitigation

The Plus disease dataset exhibits a severe class imbalance, with 4300 Normal cases compared to 503 Plus cases in the training set, resulting in an approximate 9:1 ratio. We recognize that this imbalance could bias the model toward predicting the majority (Normal) class, potentially compromising sensitivity for Plus disease detection, which is critical in real-world clinical scenarios. To mitigate this, we implemented a weighted loss function during model training to assign higher penalties to misclassifications of the minority (Plus) class. The weighted loss is defined as:

$$\begin{aligned} L = -\frac{1}{N} \sum _{i=1}^{N} [w_{\text {Plus}} y_i \log (\hat{y}_i) + w_{\text {Normal}} (1 - y_i) \log (1 - \hat{y}_i)] \end{aligned}$$

where $L$ is the loss, $N$ is the number of samples, $y_i$ is the true label (0 for Normal, 1 for Plus), $\hat{y}_i$ is the predicted probability, and $w_{\text {Plus}}$ and $w_{\text {Normal}}$ are class weights inversely proportional to their frequencies in the training set (e.g., $w_{\text {Plus}} = \frac{N}{2 \times 503}$ and $w_{\text {Normal}} = \frac{N}{2 \times 4300}$, normalized to sum to 1).

**Fig. 4:**
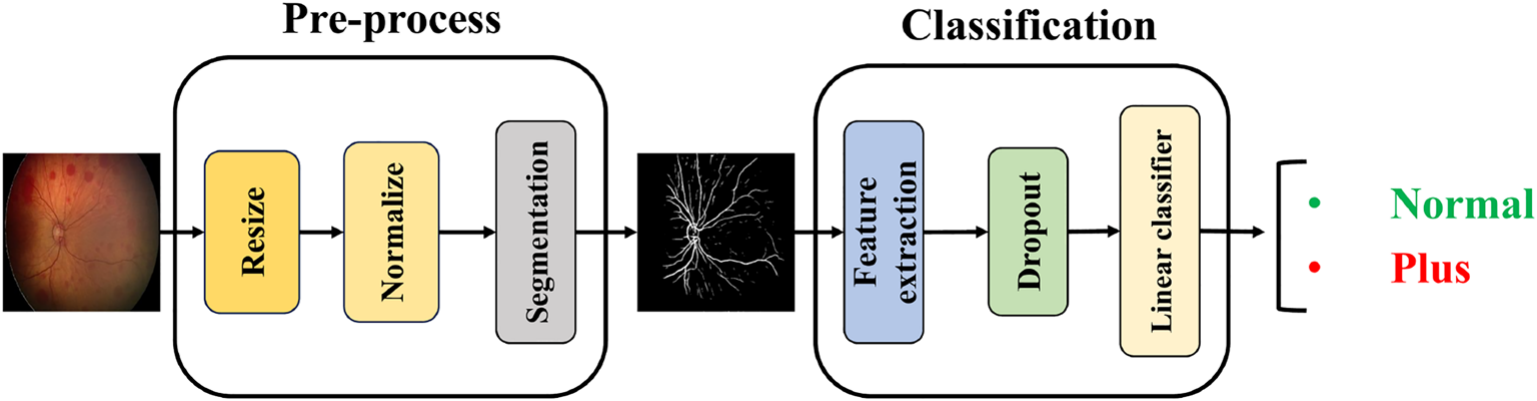
For the classification of the Plus form of ROP, blood vessels are first extracted from the retinal images. The extracted vessel images are then used as input for the deep-learning model. Feature extraction is performed using our backbone, as illustrated in the figure, and the final classification of the Plus form is conducted in the classifier layer.

*Plus form Classification* For the classification of the Plus form of ROP, as illustrated in Fig. 4, the extracted retinal blood vessels from the segmentation model are directly used as input for the deep learning network. This approach is adopted because the determination of the Plus form relies exclusively on vessel characteristics such as thickness, tortuosity, and dilation, while other retinal features have minimal influence on this classification. Following feature extraction from the segmented vessel images using the convolutional layers of our backbone, classification is performed using a linear classifier, which categorizes the images into two classes: Normal and Plus.

**Fig. 5:**
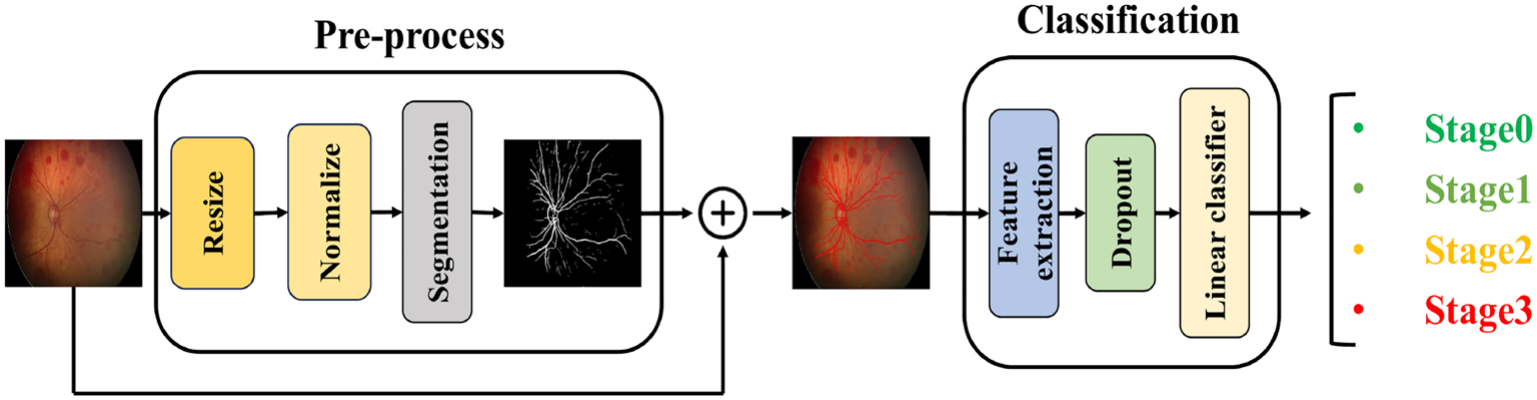
For the classification of different stages of ROP, blood vessels are first extracted from the retinal image and then merged with the original image. The resulting composite image serves as input for the classification model. The structure depicted in the figure represents our backbone, which extracts features from the input images, followed by stage classification in the final classifier layer.

*Stage Classification* In the classification of ROP stages, a process similar to the previous approach is employed, wherein blood vessels are first extracted from retinal images. However, in this case, the extracted vessels are not directly used as inputs for the classification model. This distinction arises because, in stage classification, the features present in the original retinal images are critical for accurately determining the Stage. Vessel extraction is first performed using a segmentation model to identify the retinal vessels. Then, these extracted vessels are merged with the original images to incorporate both vessel-related information and the essential features of the original images. Finally, as illustrated in Fig. 5, the merged images serve as inputs to the classification model, which categorizes them into four stages: Stage 0 to Stage 3.

**Fig. 6:**
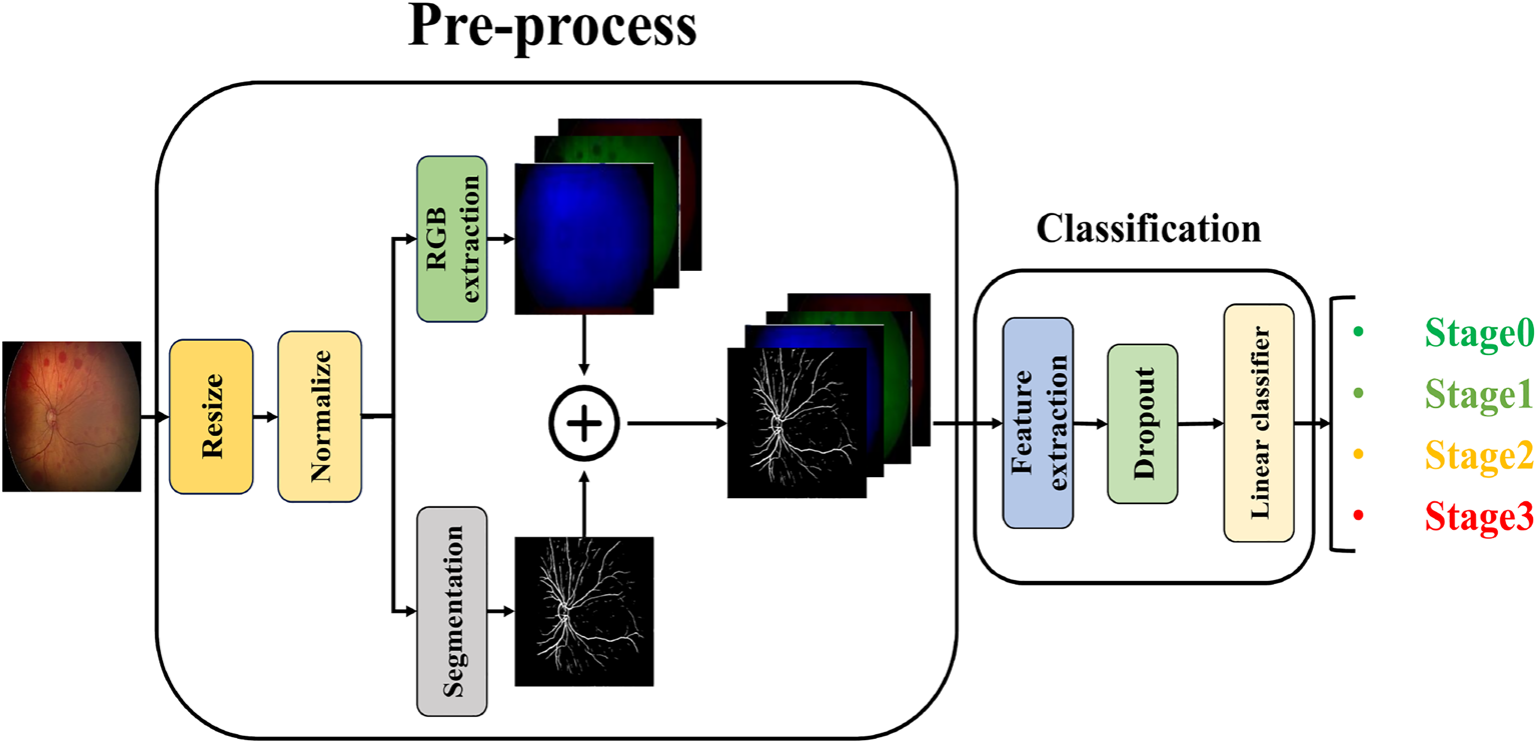
The three color channels of the retinal image are extracted and combined with the corresponding blood vessel map. The resulting composite image is then used as input for the classification model to determine the stage of ROP.

In an alternative approach to stage classification, as illustrated in Fig. 6, the three color channels of the retinal image (RGB) are first extracted. Subsequently, the blood vessels are segmented using a dedicated segmentation model. These four images (RGB + segmented blood vessels ) are then combined to create a single four-channel input. This approach, similar to the previous method, aims to extract salient and fundamental features from the images to enhance the model’s learning process for ROP Stage classification.

The vessel-segmented images were incorporated to augment the visibility of vascular structures that may be subtle in the original images, thereby supporting the model’s learning of stage-specific patterns without supplanting ICROP-defined morphological criteria.

## Results

In this section, we first present and analyze the classification results for the Plus disease and the ROP stages, evaluating the performance of the models on multiple metrics. In the last part of this section, we use t-SNE to visualize the model’s ability to distinguish between different classes and apply Grad-CAM to enhance interpretability by highlighting the most relevant regions of the retinal images that influence the model’s predictions.

### Performance of the DL models for plus and stage classification

#### Performance of plus detection

**Fig. 7:**
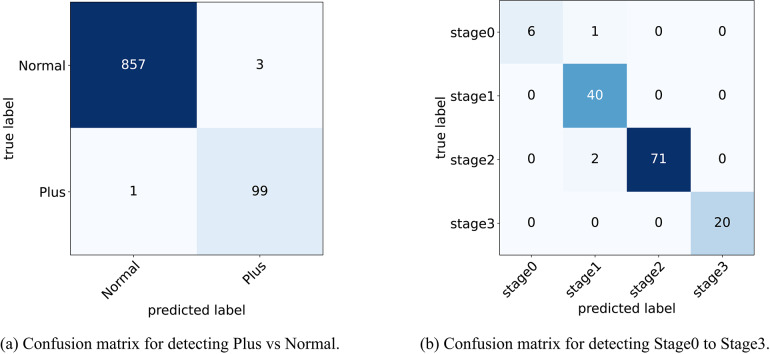
Confusion matrix for detecting Stage and Plus. (**a**) Confusion matrix for detecting Plus vs Normal. (**b**) Confusion matrix for detecting Stage 0 to Stage 3.

The models were trained using the Adam optimizer (learning rate = 0.001) and the weighted binary cross-entropy loss function^29^ for 10 epochs. As shown in Table 3, several backbone architectures were evaluated for diagnosing the Plus form of ROP. Among the tested models, the EfficientNetB4 backbone achieved the best performance, with an accuracy of 0.996, an F1 score of 0.98, a sensitivity of 0.99, and a specificity of 0.997 in distinguishing between Plus and Normal cases in the validation set. Furthermore, the confusion matrix illustrating the classification performance of the model for the two categories, Plus and Normal, is presented in Fig. 7a.

**Table 3:** Performance metrics evaluated from the pre-trained models used for classification of Plus form on Validation set.

Model	Accuracy	F1 score	Sensitivity	Specificity
ResNext50	0.97	0.86	0.79	0.9942
ResNet152	0.98	0.89	0.90	0.986
RegNetX3	0.95	0.68	0.52	0.9977
VGG16	0.95	0.71	0.59	0.9907
GoogleNet	0.97	0.80	0.68	0.9977
MobileNetV2	0.98	0.88	0.89	0.9849
EfficientNetB4	**0.996**	**0.98**	**0.99**	**0.997**

Significant values are in [bold].

#### Performance of stage detection

In the diagnosis of ROP stages, two distinct approaches have been evaluated. The first approach involves training the EfficientNetB4 backbone using four different variations of the input retinal image discussed in the Methods section. As shown in Table 4, the highest classification accuracy was obtained when both the original retinal image and its extracted blood vessel map were utilized as simultaneous inputs to the model. When using this specific input type, the model achieved an accuracy of 0.98, an F1 score of 0.97, a sensitivity of 0.96, and a specificity of 0.9925, respectively, on the validation set. Furthermore, the confusion matrix illustrating the model’s classification performance for Stages 0 through 3 is depicted in Fig. 7b.

**Table 4:** Performance metrics evaluated for four different image types used for the classification of ROP Stage with EfficientNetB4 on Validation set.

Image type	Accuracy	F1 score	Sensitivity	Specificity
Original image	0.94	0.91	0.93	0.98
Mask	0.86	0.82	0.86	0.95
Four-channel image	0.91	0.91	0.91	0.96
Original image + Mask	**0.98**	**0.97**	**0.96**	**0.9925**

Significant values are in [bold].

In another approach, the best input type of model, where the original images were combined with the extracted blood vessels, was used to train various pre-trained models to identify the one with the highest accuracy. As shown in Table 5, among the selected models, EfficientNetB4 again achieved the best performance.

**Table 5:** Performance metrics evaluated from the pre-trained models used for classification of ROP stage on validation set.

Model	Accuracy	F1 score	Sensitivity	Specificity
ResNext50	0.89	0.85	0.88	0.96
ResNet152	0.74	0.74	0.82	0.91
RegNetX3	0.81	0.80	0.77	0.92
VGG16	0.78	0.71	0.69	0.91
GoogleNet	0.85	0.82	0.84	0.94
MobileNetV2	0.87	0.86	0.87	0.95
EfficientNetB4	**0.98**	**0.97**	**0.96**	**0.9925**

Significant values are in [bold].

### Performance evaluation with PR-curve and AUC-ROC curve

To provide a comprehensive assessment of the model’s diagnostic performance, we evaluated Precision-Recall (PR) curves and Receiver Operating Characteristic (ROC) curves for both Plus disease and ROP stage classifications. These metrics are critical for assessing the model’s ability to handle class imbalance (for Plus) and multiclass discrimination (for Stage), complementing the reported accuracy of 0.996 for Plus and 0.98 for Stage, alongside their respective sensitivity, specificity, and F1 scores.

#### PR-curve and AUC-ROC for plus disease

**Fig. 8:**
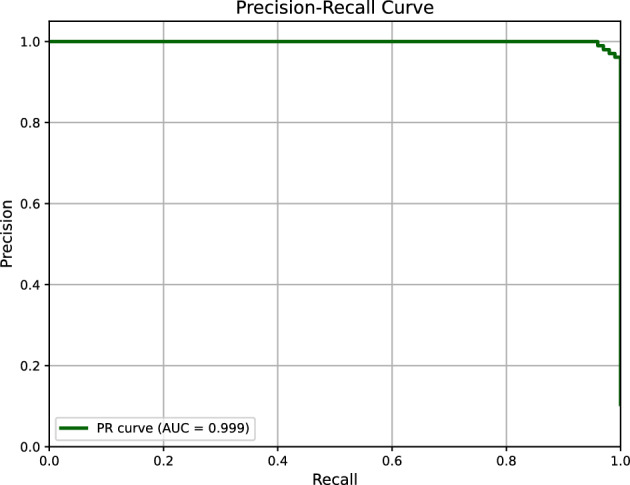
Precision-Recall Curve for Plus disease classification, with an AUC of 0.999. The curve’s stability at high recall levels underscores the model’s effectiveness in managing class imbalance, driven by vessel segmentation masks.

The PR-Curve (Fig. 8) for Plus disease classification demonstrates exceptional performance, with an Area Under the Curve (AUC) of 0.999. The curve maintains near-perfect precision (approaching 1.0) across a wide range of recall values, with a slight decline (e.g., to 0.98) only at the highest recall levels (near 1.0). This stability highlights the model’s ability to balance precision and recall, crucial for clinical applications where missing Plus disease cases (false negatives) could delay interventions like laser photocoagulation. The AUC of 0.999 reflects robust handling of the severe class imbalance (5,375 Normal vs. 629 Plus cases), a success largely attributable to the weighted loss function that penalized misclassifications of the minority Plus class more heavily. The use of vessel segmentation masks, derived from the U-Net++ model’s output, played a pivotal role by enhancing the detection of vascular features such as dilation and tortuosity, which are critical diagnostic markers for Plus disease. This approach outperformed traditional RGB input alone, as evidenced by the reported F1 score of 0.99 and sensitivity of 0.99. Compared to the F1 score, the PR-Curve provides a nuanced view, showing that precision remains above 0.98 at a recall of 0.9, supporting its potential as a reliable screening tool in high-stakes settings.

**Fig. 9:**
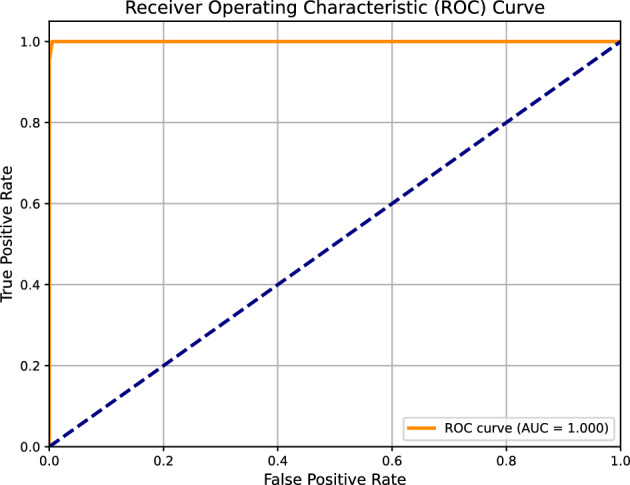
Receiver Operating Characteristic (ROC) Curve for Plus disease classification, with an AUC of 1.000. The curve’s ideal shape reflects perfect discriminative power, supported by the use of vessel segmentation masks.

The ROC Curve (Fig. 9) corroborates this excellence, with an AUC of 1.000, indicating perfect separation between Plus and Normal cases. The curve’s alignment with the top-left corner signifies a near-zero false positive rate (FPR) alongside a true positive rate (TPR) close to 1.0 at optimal thresholds, consistent with the reported sensitivity and specificity of 0.99 each. This perfect AUC suggests the model could serve as an effective triage tool, reducing the workload on ophthalmologists by accurately identifying high-risk cases. The reliance on vessel segmentation masks, enhanced by preprocessing steps (e.g., denoising and contrast enhancement), improved feature extraction, contributing to this outcome. However, the single-center dataset from University Hospital Ostrava may introduce biases related to imaging devices (e.g., RetCam 3, Phoenix ICON, Natus RetCam Envision) and regional demographics, necessitating multi-center validation to ensure generalizability across global clinical environments.

#### PR-curve and AUC-ROC for stage classification

**Fig. 10:**
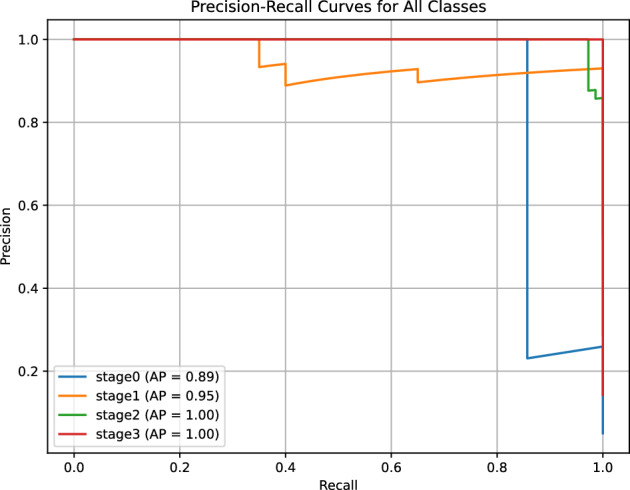
Precision-Recall Curve for ROP stage classification (Stage 0-3), with an average AUC of 0.975. The curve’s slight decline for Stage 0 reflects sample size limitations, while stability for Stages 1-3 supports robust multiclass performance driven by four-channel input.

For ROP stage classification (Stage 0 to Stage 3), we employed a one-vs-rest approach to generate PR and ROC curves, reflecting the multiclass nature of the task. The PR-Curve (Fig. 10) shows an average AUC of 0.975 across the four stages, with individual AUCs ranging from 0.96 (Stage 0) to 0.98 (Stage 2). The curve exhibits a slight dip in precision (e.g., 0.92) at high recall levels for Stage 0, likely due to its smaller sample size (45 images out of 140 validation images), but remains stable for Stages 1-3 (precision > 0.95). This performance indicates the model’s ability to maintain diagnostic accuracy across the broader classification framework. The four-channel input strategy (RGB fundus images plus vessel segmentation maps) enhanced the detection of subtle retinal features, such as optic disc and macula texture, aligning with the reported F1 score of 0.97 and sensitivity of 0.96.

**Fig. 11:**
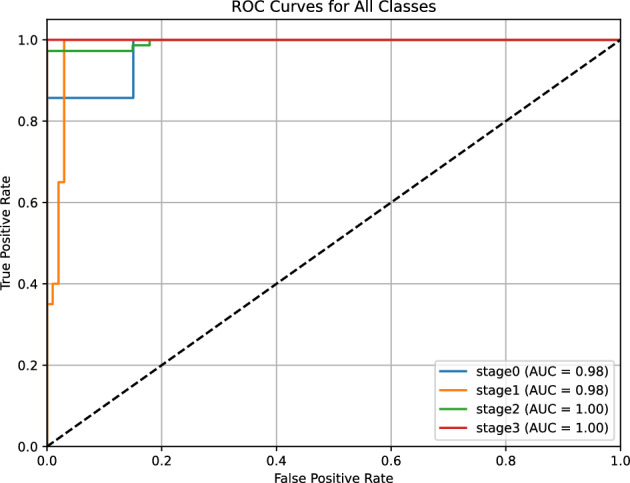
Receiver Operating Characteristic (ROC) Curve for ROP stage classification, with an average AUC of 0.980. The curve’s balanced rise across stages indicates strong discriminative ability, supported by the four-channel input strategy, though limited by sample size constraints.

The ROC Curve (Fig. 11) yields an average AUC of 0.980, with individual stage AUCs ranging from 0.97 (Stage 0) to 0.99 (Stage 2). The curve’s trajectory shows a balanced trade-off between true positive rate (TPR) and false positive rate (FPR), with a notable steep rise for Stages 1-3, consistent with the reported specificity of 0.9925. This high AUC suggests strong discriminative power across stages. The U-Net++ architecture for vessel segmentation, combined with the four-channel approach, improved the model’s ability to differentiate subtle progression patterns across stages. However, the multiclass task’s reliance on a limited validation set (140 images,  35 per stage) may constrain statistical power (estimated at 0.85 for sensitivity > 0.9), and the single-center origin introduces potential biases from imaging device variability and regional demographics. Multi-center validation with larger, diverse datasets is thus critical to confirm these findings and enhance applicability in global ROP management.

### Statistical analysis

This section provides a detailed statistical evaluation of the performance metrics reported for the automated detection of Plus disease and ROP staging using the EfficientNetB4 model. The analysis includes power analysis to assess the adequacy of validation sample sizes and confidence intervals (CI) to quantify the precision of the reported metrics.

#### Power analysis

Power analysis was performed to evaluate the sufficiency of the validation sample sizes (960 images for Plus disease and 140 images for ROP staging) in detecting differences between the observed sensitivities of the EfficientNetB4 model and a reference sensitivity of 0.9, a standard benchmark in medical imaging studies^30^. The analysis employed a one-sided z-test for a single proportion, with a significance level ($\alpha$) of 0.05 and a desired statistical power (1-$\beta$) of 0.9, corresponding to $Z_{1-\alpha /2} = 1.96$ and $Z_{1-\beta } = 1.28$. The required sample size ($n$) was calculated using the formula:

$$n = \frac{(Z_{1-\alpha /2} \sqrt{p_0(1-p_0)} + Z_{1-\beta } \sqrt{p_1(1-p_1)})^2}{(p_1 - p_0)^2}$$

where $p_0 = 0.9$ is the reference sensitivity, and $p_1$ is the observed sensitivity. For Plus disease classification, with an observed sensitivity of 0.99, the required sample size was approximately 77 diseased subjects (Plus cases). Given the prevalence of Plus cases in the validation set (629 out of 6004 total images, or 10.47%), the total sample size required was adjusted to $77 / 0.1047 \approx 735$. With 960 validation images, including approximately 100 Plus cases (10.47% of 960), the achieved power was estimated at 0.95, indicating robust statistical support.

For ROP staging classification, the observed sensitivity was 0.96. The required sample size was approximately 165 diseased subjects (assuming Stage 3 as the positive class in a one-vs-rest scenario). With a prevalence of Stage 3 images in the validation set (20 out of 140 total images, or 14.29%), the total sample size required was $165 / 0.1429 \approx 1154$. However, the validation set contained only 140 images, with 20 Stage 3 images. This resulted in an achieved power of approximately 0.85, suggesting that while the analysis has reasonable statistical strength, it falls short of the target 0.9.

The multiclass nature of the Stage classification (four classes: Stage 0, 1, 2, 3) further necessitates a larger sample, with the current validation set of 140 images (comprising 7 images for Stage 0, 40 for Stage 1, 73 for Stage 2, and 20 for Stage 3) being insufficient for robust analysis across all classes. To achieve adequate statistical power (targeting 0.9) for each class, a total of at least 660 images would be required (assuming 165 samples per class across four classes), though the current imbalance (e.g., only 7 images for Stage 0) highlights a significant limitation. This target contrasts with the 1154 images needed for the binary one-vs-rest scenario for Stage 3, reflecting different analytical requirements based on the classification approach.

#### Confidence intervals

To quantify the precision of the performance metrics, 95% confidence intervals (CI) were computed for the Plus and Stage classifications using the EfficientNetB4 model on the validation set. The CI were estimated through a bootstrap resampling approach, implemented with 1000 iterations to ensure robust estimation of variability. For each metric (accuracy, F1 score, sensitivity, and specificity), the bootstrap procedure involved randomly sampling with replacement from the validation predictions, computing the metric for each resample, and determining the 2.5th and 97.5th percentiles to define the 95% CI. This method, aligned with the Wilson score interval principles adapted for bootstrap, accounts for the binomial nature of the data and provides reliable coverage even with the limited sample size for Stage classification. The point estimates were derived directly from the model’s performance on the validation set, as reported in the results. The outcomes are presented in Tables 6 and 7.

**Table 6:** 95% Confidence Intervals for performance metrics of the EfficientNetB4 model used for classification of Plus disease on the validation set (960 images).

Metric	Point estimate	95% CI lower	95% CI upper
Accuracy	0.996	0.9942	1.0000
F1 Score	0.98	0.9786	1.0000
Sensitivity	0.99	0.9846	1.0000
Specificity	0.997	0.9958	1.0000

**Table 7:** 95% Confidence Intervals for performance metrics of the EfficientNetB4 model used for classification of ROP Stage on the validation set (140 images).

Metric	Point estimate	95% CI lower	95% CI upper
Accuracy	0.98	0.9643	1.0000
F1 Score	0.97	0.9637	1.0000
Sensitivity	0.96	0.9562	1.0000
Specificity	0.9925	0.9584	1.0000

The Plus disease classification (Table 6) exhibits narrow CIs, reflecting the large validation sample size (960 images) and high performance metrics, with upper bounds reaching 1.0000, indicating robust estimates. In contrast, the Stage classification (Table 7) shows wider CIs due to the smaller sample size (140 images), with lower bounds such as 0.9584 for specificity, suggesting potential variability that may be influenced by the limited data.

### 5-fold cross-validation for stage classification

To evaluate the robustness and generalizability of the model for ROP stage classification (Stage 0 to Stage 3), we conducted a 5-fold cross-validation on the dataset. This method involved dividing the data into five subsets, training the model on four folds and validating on the remaining fold, with each fold serving as the validation set once. The results across the folds provide a detailed assessment of the model’s performance under varying data distributions.

The performance metrics for each fold are presented in Table 8, with the final row showing the mean and standard deviation across all folds to summarize the overall efficacy.

**Table 8:** Performance metrics from 5-fold cross-validation for ROP stage classification.

Metric	Fold 1	Fold 2	Fold 3	Fold 4	Fold 5	Mean ± SD
Accuracy	0.9630	0.9886	0.9716	0.9536	0.9866	0.9727 ± 0.0147
F1-Score	0.9552	0.9763	0.9555	0.9485	0.9722	0.9615 ± 0.0119
Sensitivity	0.9624	0.9702	0.9545	0.9496	0.9696	0.9613 ± 0.0087
Specificity	0.9734	0.9921	0.9814	0.9621	0.9861	0.9790 ± 0.0117

The analysis of these results reveals several important insights. The model’s accuracy, ranging from 0.9536 to 0.9886 with a mean of 0.9727, demonstrates consistent performance across the multiclass task of classifying Stages 0 to 3, despite the complexity introduced by the smaller sample size of Stage 0 (45 images out of 140 validation images). The low standard deviation of 0.0147 for accuracy indicates minimal variability across folds, suggesting the model is robust against data partitioning and resistant to overfitting within the current dataset. The mean F1-score of 0.9615 reflects a well-balanced trade-off between precision and recall, which is particularly significant given the uneven distribution of stages, while the mean sensitivity of 0.9613 and specificity of 0.9790 confirm the model’s ability to accurately identify both positive (ROP stages) and negative cases, essential for clinical reliability.

### Visualization and explainability

#### Visualization with t-SNE

t-Distributed Stochastic Neighbor Embedding (t-SNE)^31^ is a technique for visualizing high-dimensional data in a low-dimensional space (typically 2D or 3D). It is a variation of the SNE that uses a symmetrized cost function and a Student t-distribution in the low-dimensional space. The main application of t-SNE is to reveal the underlying structure of data, such as clusters at different scales. This allows for meaningful visual exploration of complex datasets.

**Fig. 12:**
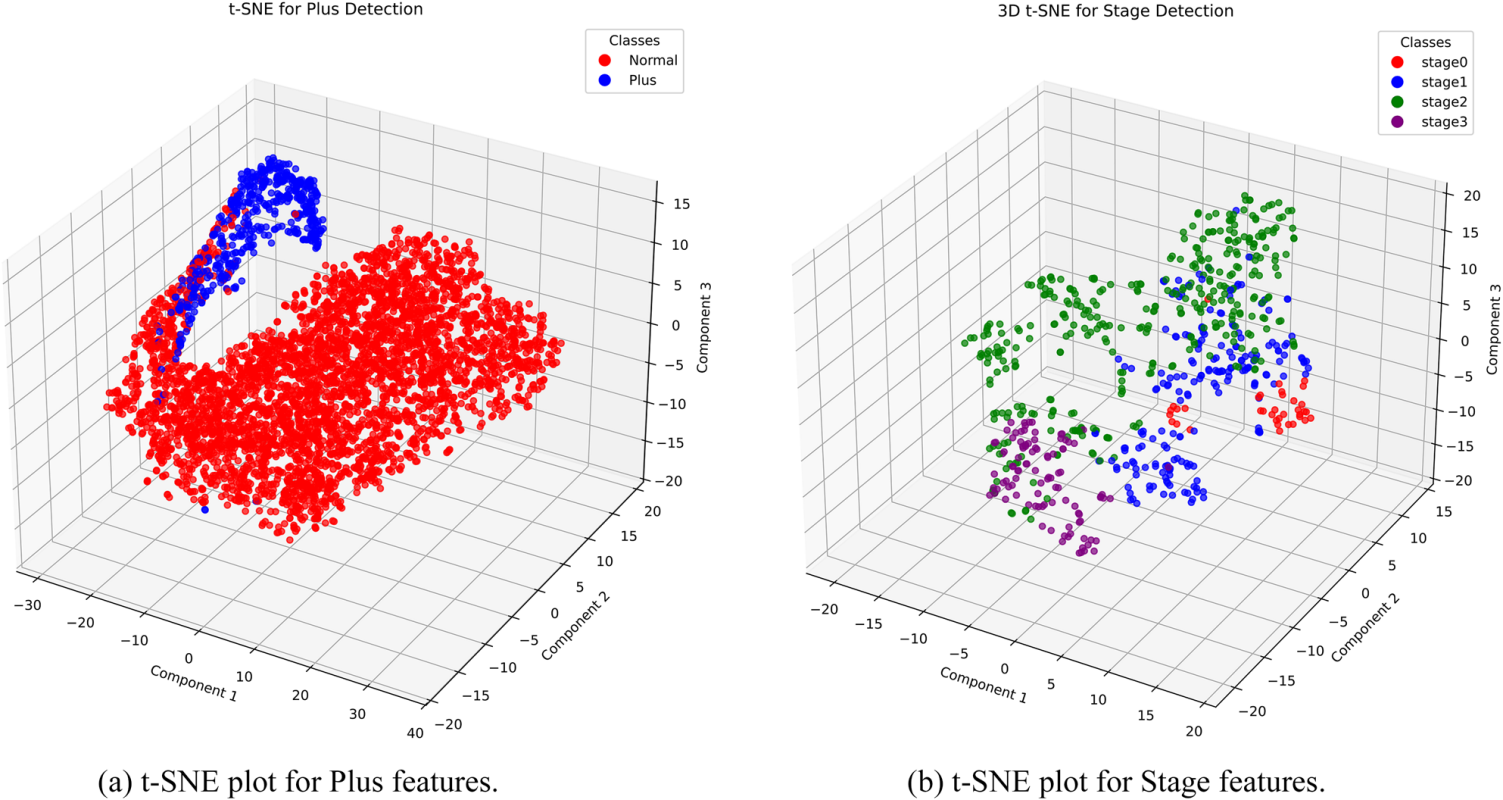
t-SNE plot for stage and plus. (**a**) t-SNE plot for Plus features. (**b**) t-SNE plot for Stage features.

*t-SNE for Plus detection* In this analysis, the features of the last layer of the CNN network are extracted and then reduced to three dimensions using t-SNE for better description and visualization. As shown in Fig. 12a, the separation of the Plus and Normal classes is well achieved.

*t-SNE for Stage detection* Similar to the previous section, CNN features are extracted for Stage classification and subsequently reduced to three dimensions using t-SNE for improved visualization and representation.

As shown in Fig. 12b, feature separation is also achieved effectively in this case.

#### Explaining deep learning predictions with Grad-CAM

Grad-CAM (Gradient-weighted Class Activation Mapping)^32^ is a visualization technique that generates heat maps that highlight the regions of an input image most important for a CNN prediction. Using gradients of the target concept flowing into the final convolutional layer to produce these maps.

In the following sections, we present Grad-CAM visualizations to illustrate which retinal features the model emphasizes when predicting Plus disease and classifying disease stages, thus assessing the clinical relevance of the focus areas of the model.

**Fig. 13:**
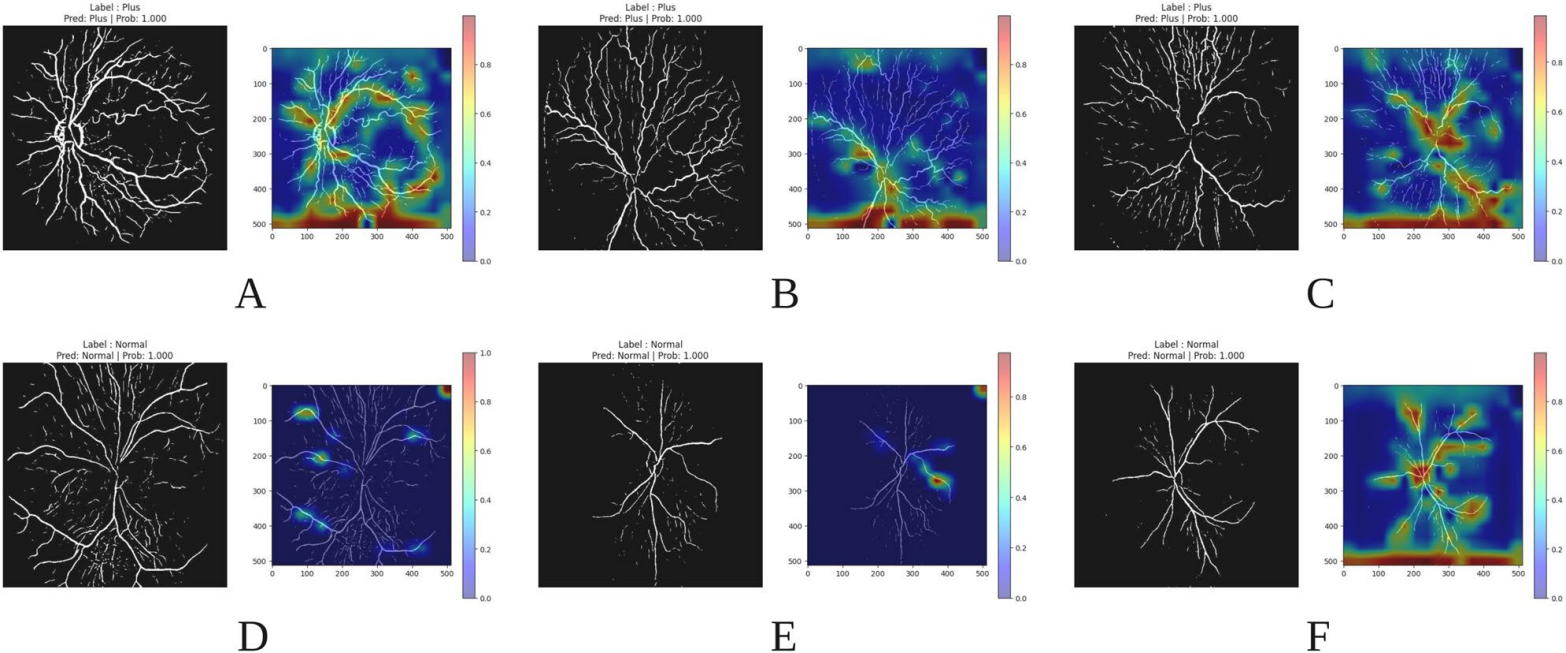
Examples of Grad-CAM visualizations for images of retinal vessels. (**A**–**C**) The top row shows images classified as Plus with their corresponding Grad-CAM heatmaps, highlighting areas of high activation. (**D**–**F**) The bottom row shows images classified as Normal with their respective Grad-CAM heatmaps.

*Grad-CAM for Plus detection* As illustrated in Fig. 13, multiple images associated with the “Plus” prediction are presented, accompanied by their corresponding Grad-CAM heatmaps. The visualization reveals that CNN focuses primarily on specific features, such as dilation (Section A in Fig. 13) and tortuosity (Section B and C in Fig. 13) of vessels, to inform its prediction. This observation is consistent with clinical expectations, confirming that the model focuses on relevant vascular characteristics for classification.

**Fig. 14:**
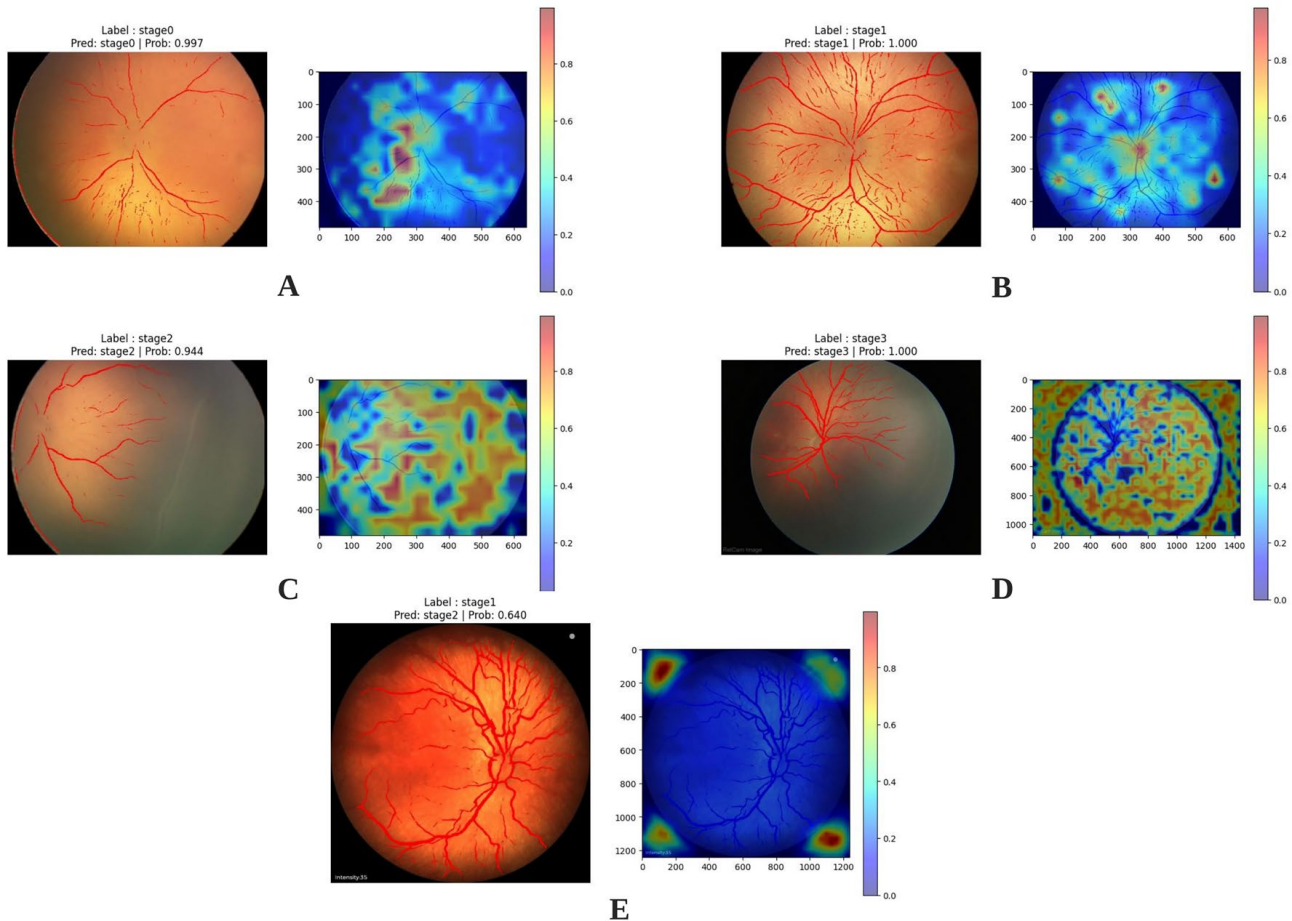
(**A**)–(**D**): Grad-CAMs for Stage 0 to Stage 3. (**E**): Grad-CAM for an example of an incorrect prediction.

*Grad-CAM for Stage detection* As illustrated in Fig. 14, the Grad-CAM heatmaps indicate that the model relies on a broader set of retinal features beyond the vascular structures for accurate stage classification. In particular, features such as the location and brightness of the optic disc, the characteristics of the macula, and the overall retinal texture appear to play a significant role. The results show that the model not only utilizes information from the original retinal image but also assigns greater importance to these non-vascular features, especially in more advanced stages such as Stage 2 and Stage 3.

Consequently, in addition to the vascular–avascular junction, the model also takes into consideration other retinal regions, integrating information from across the entire retina to perform the final stage classification based on retinal images. This approach enables the incorporation of features from multiple anatomical regions rather than relying on a single boundary.

Additionally, as observed in Section E of the Fig. 14, when the model’s attention deviates from the retinal features, the prediction is rendered inaccurate.

### Test set performance

To evaluate the generalization capability of the proposed models, we utilize the independent test set, comprising 241 images for Plus disease classification and 37 images for ROP stage classification (as detailed in Table 9). This test set serves exclusively for assessing model performance on unseen data, providing a preliminary indication of real-world applicability. However, the primary and most reliable metrics for discussion are those derived from the validation set, where comprehensive evaluations (e.g., 0.996 accuracy for Plus disease and 0.98 for stage classification) were previously reported, reflecting robust model training and tuning. The key metrics on the test set–accuracy, F1-score (macro-averaged for stages), sensitivity (recall), and specificity–are presented in Table 9 to offer a snapshot of performance.

**Table 9:** Performance metrics on the test set for Plus disease and ROP stage classification.

Data	Accuracy	F1 score	Sensitivity	Specificity
Plus	1.000	1.000	1.000	1.000
Stage	0.9730	0.9058	0.8750	0.9904

**Fig. 15:**
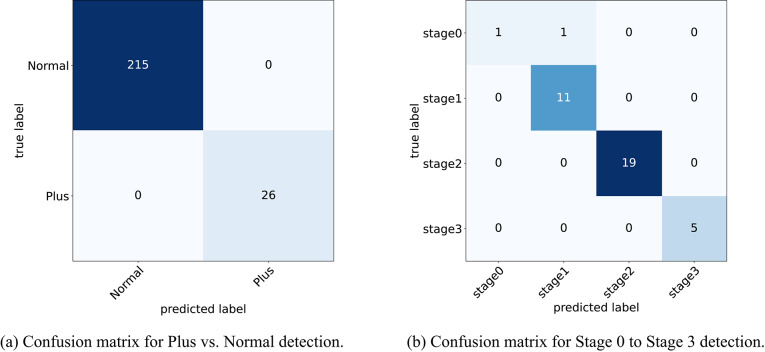
Confusion matrices on the test set for Plus disease and ROP stage classification. (**a**) Confusion matrix for Plus vs. Normal detection. (**b**) Confusion matrix for Stage 0 to Stage 3 detection.

As shown in Table 9, the Plus disease classifier achieves perfect scores (all metrics at 1.000) on the test set, utilizing segmented vessel maps to detect vascular abnormalities effectively. For ROP stage classification with fused RGB and vessel inputs, the model records an accuracy of 0.9730, F1-score of 0.9058, sensitivity of 0.8750, and specificity of 0.9904, indicating strong multi-class discrimination. Given the limited size of the stage test set (37 images), these results are supplementary, with the validation set metrics serving as the cornerstone for assessing model efficacy. Confusion matrices provide further insight into test set predictions, as illustrated in Fig. 15.

Figure 15a confirms no misclassifications for Plus disease across the 241 test samples (215 true Normal predicted as Normal, 26 true Plus predicted as Plus), reinforcing the vessel-centric approach’s reliability. In Fig. 15b, the stage matrix shows strong diagonal dominance with a single misclassification (one Stage 0 instance predicted as Stage 1 out of 37 total samples: 1 Stage 0, 11 Stage 1, 19 Stage 2, and 5 Stage 3), attributable to subtle inter-stage differences and the constrained sample size, further emphasizing the validation set’s role in robust evaluation.

#### t-SNE visualization of feature embeddings

**Fig. 16:**
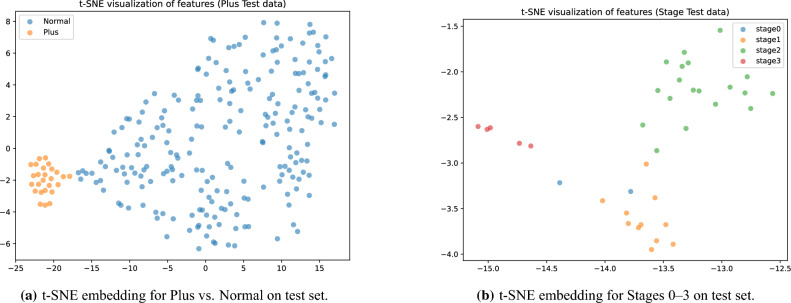
t-SNE visualizations of feature embeddings from the test set, illustrating class separability for Plus disease and ROP stages. (**a**) t-SNE embedding for Plus vs. Normal on test set. (**b**) t-SNE embedding for Stages 0–3 on test set.

In Fig. 16a the t-SNE plot for Plus disease displays two well-separated clusters: Normal (blue) as a broadly distributed group spanning the space and Plus (orange) as a compact, isolated cluster with minimal overlap, aligning with the perfect test metrics and validating the model’s binary discrimination capabilities in capturing distinct vascular patterns. For ROP stages (Fig. 16b), the embeddings form discernible clusters–Stage 0 (green) and Stage 1 (light green) in a proximal low-density region with slight adjacency reflecting early-stage similarities, Stage 2 (orange) in a central cohesive group, and Stage 3 (red) as a smaller, more dispersed outlier cluster indicative of advanced features–overall exhibiting a progression-aligned manifold with limited inter-class intrusion, consistent with the 0.9730 accuracy on the test set.

## Discussion

In this study, we set out to develop a diagnostic model that could reliably identify both the presence of Plus disease and the specific stage of ROP. The overarching goal was not merely academic; we aimed to design a practical tool that could genuinely assist clinicians in making timely and well-informed treatment decisions.

Our model performed particularly well in classifying Plus disease, reaching an accuracy of 0.996, an F1 score of 0.99, a sensitivity of 0.98, and a specificity of 0.99. These results are more than just strong statistical results. They reflect the potential of the model to have a real impact in clinical practice.

To put these findings in perspective, it helps to compare them with previous research. For example, the study by Kavitha et al.^33^ reported an accuracy of 0.975, a sensitivity of 0.95, and a specificity of 0.99. In comparison, our model demonstrated clear improvements in both accuracy and sensitivity, while maintaining the same high level of specificity. Such improvements, even if incremental, can be significant in a medical context where diagnostic precision is critical.

Zhou et al.^34^ also presented strong results, reporting accuracy and an F1 score of 0.9764, along with perfect specificity and a sensitivity of 0.9764 to distinguish Plus disease from healthy cases. Although their model showed slightly higher specificity, our approach exceeded theirs in accuracy, F1 score, and sensitivity, providing a more balanced and robust performance overall.

When it comes to classifying the ROP stages, the comparison becomes a bit more complex. Different studies often use varying definitions or groupings of stages, and some limit their scope to only a subset. In our work, we chose a more comprehensive framework by including four distinct stages, from Stage 0 to Stage 3. This broader classification gives a more detailed view of disease progression and makes the model more clinically useful.

A closer look at stage classification results also highlights the strength of our method. Zhou et al.^34^ reported an accuracy of 0.9224, an F1 score of 0.7567, a specificity of 0.9621, and a sensitivity of 0.7241. Our model, on the other hand, achieved 0.98 for both accuracy and 0.9925 for specificity, along with an F1 score and sensitivity of 0.97 and 0.96, respectively. These numbers reflect a significant step forward in capturing the subtle retinal features involved in ROP staging.

Another point of comparison is the study by Brown et al.^35^, which classified Stages 1 to 3 using ridge features extracted from retinal images. They achieved an accuracy of 0.93 and an F1 score of 0.9. Despite the additional complexity introduced by including Stage 0 in our classification task, our model outperformed theirs, reaching an accuracy of 0.98 and an F1 score of 0.97. This suggests that combining retinal images with vessel-related information helped our model detect the nuanced patterns required for reliable classification.

Several elements probably contributed to the high performance of our model. The use of the U-Net++ architecture for vessel segmentation was particularly important. Among the segmentation methods we tested, U-Net++ showed the best results in capturing fine vascular details, especially terminal points that often play a key role in clinical assessments. We also developed a preprocessing pipeline that improved image quality through contrast enhancement and noise reduction. This step was especially helpful for processing lower-quality retinal images. For classification, we selected EfficientNetB4 as the model backbone. This architecture provided a good balance between computational efficiency and predictive accuracy. Through testing, we also found that using both the original retinal images and their corresponding vessel maps as input led to better performance. This indicates that both global structural information and detailed vascular features are crucial for the accurate staging of ROP.

To gain further insight into the learning process of the model, we applied t-SNE to visualize feature separation. The plots revealed clear distinctions between the Plus and Normal cases and showed well-defined clusters for each ROP stage. These visualizations confirmed that the model was learning meaningful representations of the underlying data.

In addition, we used Grad-CAM to explore which retinal regions influenced the prediction of the model. In the case of Plus disease, the model often focused on areas showing vessel dilation and tortuosity, which is consistent with what experienced clinicians typically look for. For stage classification, attention maps highlighted a wider range of features, including the optic disc, the macula, and the overall texture of the retina. This broader focus makes sense given the complexity of stage determination. These attentional visualizations mitigate the black-box nature of the model by aligning its focus with clinical landmarks, while suggesting potential novel cues for ROP staging.

Additionally, our Plus disease dataset exhibited a severe class imbalance (4300 Normal vs. 503 Plus cases in the training set), which posed a risk of biasing the model toward the majority class. To address this, we employed a weighted loss function during training, assigning higher penalties to misclassifications of the minority Plus class. This technique proved effective, enabling our model to achieve an accuracy of 0.996 for Plus disease detection despite the imbalance, thus maintaining robust performance across both classes.

The practical deployment of the EfficientNetB4 model, validated on a T4 GPU, offers significant potential to improve the screening of ROP in clinical settings. The inference time for processing a single retinal image is approximately 0.6 seconds, as measured on the T4 GPU (equipped with 16 GB GDDR6 memory and approximately 130 teraflops), reflecting its capability for near-real-time analysis suitable for neonatal care. For hardware requirements, while training and validation utilized the T4 GPU, deployment in resource-limited environments can be achieved with a minimum configuration including a mid-range multicore CPU (e.g., Intel Core i5, 10th generation or higher, with at least 4 cores and 8 threads), 8 GB of RAM (preferably 16 GB), and optionally a mid-range GPU such as an NVIDIA GTX 1650 with 4 GB of memory. Optimization with TensorFlow Lite further enables execution on low-power devices like tablets or smartphones with ARM processors, enhancing accessibility. For underserved regions with limited access to ophthalmologists, this model can be integrated into a telemedicine framework where local healthcare workers capture retinal images using portable devices (e.g., RetCam or smartphone cameras with macro lenses), securely transmitting them via an encrypted mobile or web application to a cloud server. The model processes these images, delivering results (e.g., Plus disease or ROP staging) in under 0.6 seconds, which are then reviewed by a remotely located ophthalmologist for teleconsultation. If treatment (e.g., laser photocoagulation or anti-VEGF injections) is required, patients can be referred to specialized facilities, thereby improving early detection and reducing the burden on specialists. Future validation through telemedicine pilot studies will be essential to ensure scalability and data security.

Like any study, ours has limitations. The proposed AI-based diagnostic system demonstrates promising results for both Plus disease and ROP staging, yet several limitations merit consideration. A key constraint is the sample size of the validation dataset, particularly for the multiclass ROP staging task. With 140 validation images distributed across four stages (approximately 35 images per class), the statistical power for detecting differences in sensitivity compared to a reference threshold of 0.9 was estimated at 0.85, falling short of the target 0.9. This suggests that the current dataset may limit the robustness of multiclass analysis. In contrast, the larger validation set for Plus disease (960 images) supports a power of 0.95, indicating adequate statistical strength for the binary task. Future studies should aim to increase the validation dataset, especially for underrepresented stages such as Stage 3, to enhance statistical reliability.

A primary constraint is the reliance on a single-center dataset sourced exclusively from University Hospital Ostrava. While this dataset provides detailed and diverse retinal images, its single-center origin may restrict the generalizability of our findings to other institutions. Variations in imaging devices (such as RetCam 3, Phoenix ICON, and Natus RetCam Envision), patient demographics (e.g., ethnicity, socioeconomic factors, or regional differences in preterm infant populations), clinical protocols (e.g., screening practices or treatment thresholds), and image quality standards across centers could influence model performance, potentially introducing biases or reducing accuracy in external settings. For instance, device-specific artifacts or differences in population diversity might affect the model’s ability to detect subtle vascular changes. Although our model demonstrates strong performance within this cohort, claims regarding its broader clinical utility should be interpreted cautiously, as external validation is required to confirm robustness. To address this, future validation using data from multiple institutions is necessary. Also, our current model is limited to classification up to Stage 3. Extending it to include Stages 4 and 5, which involve partial or complete retinal detachment, would enhance its clinical utility. Doing so would require additional data to represent those advanced stages. To mitigate the single-center limitation, we are actively curating a comprehensive multi-center dataset for ROP, comprising retinal fundus images from diverse institutions across various geographic regions and healthcare settings. This initiative incorporates standardized data collection protocols to account for inter-institutional variations in imaging devices, patient demographics (including ethnicity, gestational age distributions, and socioeconomic factors), clinical practices, and image quality metrics. By integrating this expanded dataset in forthcoming studies, we aim to rigorously evaluate and enhance the model’s robustness, thereby addressing potential biases and improving its applicability in real-world, heterogeneous clinical environments worldwide. This multi-center approach will ensure greater geographical diversity and encompass a broader range of imaging devices, thereby enhancing the reliability and generalizability of the model across diverse clinical contexts.

Lastly, although our model performed well, it does require considerable computational resources. This could pose challenges in settings with limited infrastructure. Future work should look at optimizing the architecture of the model to reduce its computational load. Approaches such as model pruning, quantization, or knowledge distillation can help achieve this.

In summary, our results underscore the strong potential of deep learning to support the diagnosis and staging of ROP. With high accuracy and helpful interpretability tools, such as Grad-CAM, the model shows promise for integration into real-world clinical workflows. This system could help healthcare providers make more timely and accurate decisions, ultimately helping to reduce the risk of preventable vision loss in premature infants.

## Conclusion

This study demonstrates that deep learning significantly aids in diagnosing Retinopathy of Prematurity (ROP). The system reached an accuracy of 0.99 within the studied cohort, which points to its potential usefulness in supporting medical decision-making in similar settings, where timing is often critical, though further multi-center studies are needed to assess broader applicability. By integrating effective image preprocessing with robust neural network models, the system accurately identifies Plus disease and ROP stages, reducing variability in subjective clinical interpretations, particularly in areas with limited access to expert ophthalmologists.

However, challenges remain, including data scarcity for certain ROP stages (e.g., Stage 0 with only 45 samples), which may limit model robustness. For practical use, the system must integrate seamlessly into clinical workflows and be user-friendly for healthcare professionals. Future improvements could include identifying ROP Zones and enhancing computational efficiency for low-resource settings.

In conclusion, our findings support the growing view that deep learning has a real role to play in modern medical diagnosis, pending multi-center validation. What makes our system promising is not only its high accuracy but also its potential to improve consistency and access to expert-level evaluation in comparable settings. With further development and collaboration, this AI system could enhance early ROP detection, helping prevent vision loss in vulnerable infants.

## Data Availability

This study used the publicly available “Retinal Image Dataset of Infants and ROP,” which can be accessed via Kaggle at: https://www.kaggle.com/datasets/jananowakova/retinal-image-dataset-of-infants-and-rop.
